# Corrected Scoliosis, Cholinesterase Deficiency, and Cesarean Section: A Case Report

**DOI:** 10.1155/2009/957479

**Published:** 2009-06-23

**Authors:** Roy Somers, Yves Jacquemyn, Luc Sermeus, Marcel Vercauteren

**Affiliations:** ^1^Department of Anaesthesiology, Antwerp University Hospital (UZA), Wilrijkstraat 10, 2650 Edegem, Belgium; ^2^Department of Obstetrics and Gynaecology, Antwerp University Hospital (UZA), Wilrijkstraat 10, 2650 Edegem, Belgium

## Abstract

We describe a patient with severe scoliosis for which corrective surgery was performed at the age of 12. During a previous caesarean section under general anaesthesia pseudocholinesterase deficiency was discovered. Ultrasound guided spinal anaesthesia was performed enabling a second caesarean section under loco-regional anaesthesia.

## 1. Introduction

Pseudocholinesterase deficiency is an enzymatic abnormality resulting in abnormally slow metabolic degradation of exogenous choline-ester drugs such as succinylcholine. This requires prolonged ventilation with concommitant sedation. This is not always appreciated by the patient especially not by parturients with the desire to wake up as soon as possible to take care of their neonate. If combined with factors endangering the success of loco-regional anaesthesia, the management of such a patient becomes a challenge.

## 2. Case Report

**Figure 1:**
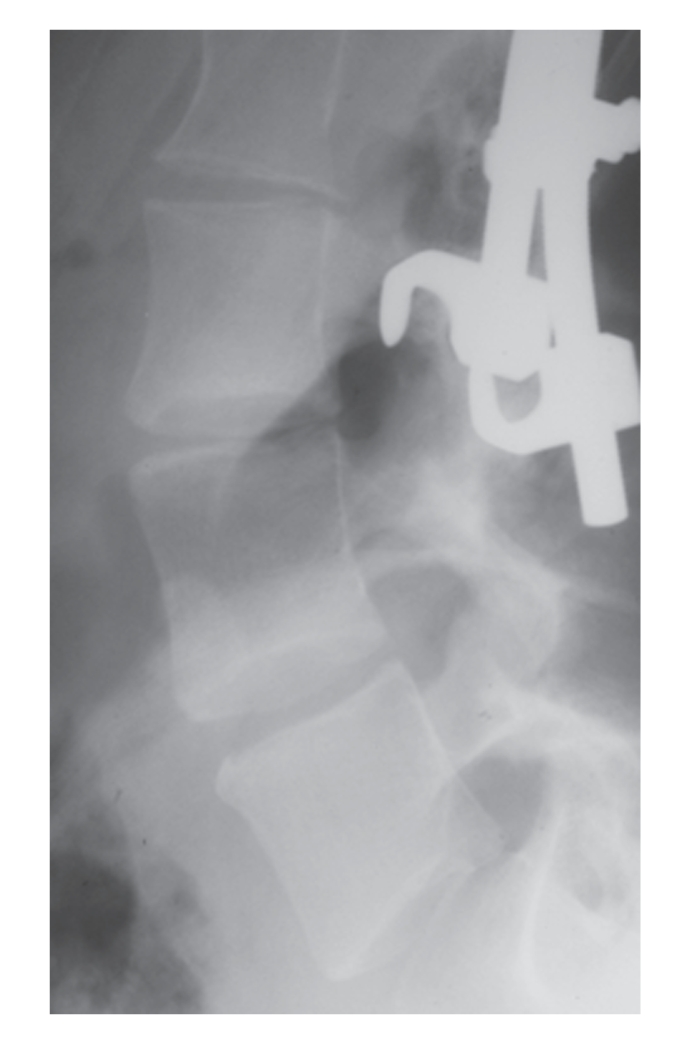
Radiography of the lumbal spine demonstrating osteosynthetic material until the border of L3.

A 30-year-old parturient was born with congenital scoliosis, necessitating repeat surgical procedures in childhood and vertebral fusion osteosynthesis, extending up to the vertebral body of L4 (Figure 1) at the age of 12. In her first pregnancy CT pelvimetry was performed, demonstrating major pelvic deformity and it was opted to perform a caesarean section under general anaesthesia at a gestational age of 39 weeks. General anaesthesia was induced after preoxygenation, with thiopental and succinylcholine intravenously, and tracheal intubation was easily performed under cricoid pressure.

The surgical procedure was uneventful and a healthy daughter was born with Apgar scores 9 and 9 at 1 and 5 minutes, respectively. At the end of the procedure the patient did not return to spontaneous breathing due to prolonged neuromuscular blockade, and cholinesterase deficiency was suspected. She remained mechanically ventilated for 6 hours after which she resumed spontaneous respiration. Postoperatively cholinesterase was determined in serum with spectophotometry (Vitros 5.1 FS Ortho Clinical Diagnostics) and the value was 1123 U/L (normal 4650–10440 U/L). There were no other complications in the postpartum period.

She consulted again for a second pregnancy three years later. No levels of cholinesterase in the interval between pregnancies were available. At a gestational age of 32 weeks serum cholinesterase level was 1479 U/L. It was decided to try spinal anaesthesia with placement of the needle under ultrasound guidance at the lumbar 4th–lumbal 5th level. At gestational age 39 weeks a repeat caesarean section was planned. As a preload 500 mL, 6% polyhydroxyethylstarch (Voluven, Fresenius Kabi, The Netherlands) was given. Ultrasound (Logiq-E, General Electrics with a 12 MHZ probe) was performed to locate the lower boarder of the osteosynthetic material and after ultrasound determination of the depth of the dura mater and localization of the intervertebral space, a 27-Gauge Whitacre Spinal Needle (Becton Dickinson, Madrid, Spain) was introduced successfully at first attempt in the lumbar interspace L5-S1 and 10 mg levobupivacaine (5 mg/mL) with 5 *μ*g sufentanil (5 *μ*g/mL) was injected in the subarachnoid space. An upper sensory level of T5 was obtained. An uneventful repeat caesarean section was performed and a healthy girl, weighing 3.810 g with Apgar scores of 8 and 9 after 1 and 5 minutes, respectively, was born. Umbilical artery pH value was 7.35. The postoperative period was without any complications.

## 3. Discussion

Pseudocholinesterase deficiency is an autosomal recessive disorder (OMIM 177400, synonyms: butirylcholinesterase deficiency, suxamethomiun sensitivity), different mutations of the gene located on chromosome 3q26 have been reported [1]. We did not identify the specific mutation in our patient as this would not influence the clinical management. Pseudo cholinesterase is a glycoprotein produced by the liver, circulating in the plasma. It does not have any known physiologic function, but a detoxifying role has been suggested. Pseudo cholinesterase deficiency results in not only abnormally slow degradation of exogenous cholinester drugs such as succinylcholine and some nondepolarizing muscle relaxants such as mivacurium but also the degradation of procaine and cocaine. 

The condition is recognized most often when respiratory paralysis unexpectedly persists for a prolonged period of time following administration of succinylcholine. The mainstay of treatment is ventilatory supported until diffusion of succinylcholine out of the myoneural junction permits return of neuromuscular function. The diagnosis is confirmed by a laboratory assay demonstrating decreased plasma cholinesterase enzyme activity. Another proposed treatment is prophylactic transfusion of fresh frozen plasma which can augment the patients endogenous plasma pseudocholinesterase activity. This practice is not recommended because of the risk of iatrogenic viral infectious complications. However, perioperative transfusion of fresh frozen plasma administered to correct a coagulopathy may mask an underlying pseudo cholinesterase deficiency [1]. 

Different authors have demonstrated lower plasma cholinesterase activity in pregnant patients as compared to nonpregnant women. Blitt et al. could not demonstrate a correlation between plasma cholinesterase activity and duration of paralysis form succinylcholine [2] although others did [3, 4]. During the first 2 to 3 days postpartum a further fall in cholinesterase activity has been described followed by a rise to normal levels by the end of the puerperium [5]. Lower levels of cholinesterase have been noticed in case of pre-eclampsia and haemolysis, elevated liver tests, and low platelets (HELLP) syndrome, probably due to diminished liver function [6]. 

We have been able to find 13 reports on cholinesterase deficiency during caesarean section [3, 4, 6–16]. Two cases of epidural anesthesia with chloroprocaine complications such as high epidural block with respiratory problems and grand mal seizures have been reported [15, 17]. Five cases have been described with transient respiratory depression of the newborn when the mother had cholinesterase deficiency, probably demonstrating the baby being heterozygous for the allele and receiving succinylcholine through placental transfer [18, 19].

Remifentanil is a rapidly metabolised opioid *μ*-receptor agonist and had been suggested as a suitable alternative to neuromuscular blocking drugs to facilitate tracheal intubation. Should mask ventilation become necessary, in case of remifentanyl, muscle rigidity might make this more difficult. Remifentanyl has been used for caesarean section [20] and a case has been reported in a patient with a history of multiple spinal operations and scoliosis combined with cholinesterase deficiency [16]. In this case tracheal intubation was easily possible and the patient awoke immediately after surgery, the neonate had normal Apgar scores and umbilical cord blood gases. We have been able to find only one report of an obstetric patient with Harrington rods where ultrasound was used to perform epidural analgesia for labour and vaginal deliçvery, not for caesarean section [21].

We conclude that in patients with pseudo cholinesterase deficiency and difficulties for loco-regional anaesthesia ultrasound guided puncture can provide a possibility for loco-regional anaesthesia previously not possible, in a setting with facilities for ultrasound guided needle placement.
